# Supplementary material for: A do-it-yourself 3D-printed thoracic spine model for anesthesia resident simulation
Source: PLoS One. 2020 Mar 11;15(3):e0228665. doi: 10.1371/journal.pone.0228665 (PMC7065759; doi:10.1371/journal.pone.0228665)
Supplement: S2 File — (PDF) [file pone.0228665.s002.pdf]

The authors declare no conflict of interest.
This work was supported by departmental funding.

REFERENCES

1. Wiegel M, Moriggl B, Schwarzkopf P, Petroff D, Reske AW. Anterior suprascapular nerve block versus interscalene brachial plexus block for shoulder surgery in the outpatient setting: a randomized controlled patient- and assessor-blinded trial. *Reg Anesth Pain Med*. 2017;42:310–318.
2. Abdallah FW, Halpern SH, Aoyama K, Brull R. Will the real benefits of single-shot interscalene block please stand up? A systematic review and meta-analysis. *Anesth Analg*. 2015;120: 1114–1129.
3. Falcão LF, Perez MV, De Castro I, Yamashita AM, Tardelli MA, Amaral JL. Minimum effective volume of 0.5% bupivacaine with epinephrine in ultrasound-guided interscalene brachial plexus block. *Brit J Anaesth*. 2013;110: 450–455.
4. Riazi S, Carmichael N, Awad I, Holtby RM, McCartney CJ. Effect of local anaesthetic volume (20 vs 5 mL) on the efficacy and respiratory consequences of ultrasound-guided interscalene brachial plexus block. *Br J Anaesth*. 2008;101:549–556.

Reply to Dr Zhou et al

Accepted for publication: September 2, 2017.

To the Editor:

Zhou et al[1] take issue with our trial that compares the interscalene brachial plexus block with the suprascapular nerve block (SSNB)[2] and conclude that "future studies should examine clinically important outcomes." We could not agree more and were pleased to see that the authors are near completion of a trial assessing postoperative pain severity (NCT02517437), while we are currently recruiting for a trial to detect impaired ventilation as a result of phrenic nerve blocks (DRKS00011787).

The authors raise 3 points: the validity of our measure of pain control, the fairness of comparing 20 mL ropivacaine (interscalene brachial plexus block) versus 10 mL (SSNB), and the clinical relevance of the motor block.

Regarding pain (Figure 3 in our article[2]), one can debate whether procedural pain should be included, but considering that this accounts for only 0.3% of the area under the curve, it plays essentially no role in the primary outcome. We certainly agree that more time points for measuring pain would have been preferable, but unfortunately not feasible in our outpatient setting. However, the numerical rating scale values for pain are so similar between the groups at each point in time after the operation that noninferiority is essentially guaranteed, independent of a potential rebound effect. The mean numerical rating scale score between the 4th and 24th hour for the SSNB group would have to be 3.6 instead of 2.0 for noninferiority no longer to hold, a value 3 SEs larger than the interpolated one and so large that it would extend beyond the scale of the figure.

Regarding grip strength, the authors contend that SSNB was favored from the outset because of our choice of anesthetic volumes. There is, of course, some merit to this argument, and in our article, we explained that the choice was based on some of the lowest volumes used at the time. We elaborated on this point in response to another letter to the editor,[3] noting that with increasing experience, volumes can be reduced. Moreover, the duration of the block, not only its initial success, has to be taken into account. In the active trials mentioned previously, we are using 10 mL of 1% ropivacaine, and the authors of the letter used 20 mL of 0.5% ropivacaine. As one reduces volumes below, say, 10 mL, the success of the block will depend increasingly on the experience and skill of the anesthesiologist, and the generalizability of publications may have to be scrutinized closely.

The authors finally question the relevance of looking at motor blocks of the hand, given that "most shoulder patients are immobilized in slings for several weeks." For one thing, more than 25% of the patients we studied were not in fact immobilized (Table 1 in our article[2]). Moreover, ability to use the hand despite immobilization is important for daily routine such as use of mobile phones, drinking, or personal hygiene.

There are certainly a number of clinical issues that still need to be addressed, and we are looking forward to reading upcoming publications from the trials that are underway.

Martin Wiegel, MD
Department of Anesthesiology
ACQUA Clinic
Leipzig, Germany

David Petroff, PhD
Clinical Trial Centre
University of Leipzig
Leipzig, Germany

Andreas W. Reske, MD, PhD
Department of Anesthesiology
and Intensive Care Medicine
Heinrich-Braun-Hospital
Zwickau, Germany
and Department of Anesthesiology
and Intensive Care Medicine
University Hospital Leipzig
Leipzig, Germany

The authors declare no conflict of interest.

REFERENCES

1. Zhou C, Choi S, Brull R, Abdallah FW. Anterior suprascapular nerve block versus interscalene brachial plexus block for shoulder surgery in the outpatient setting. *Reg Anesth Pain Med*. 2018; 43:99–100.
2. Wiegel M, Moriggl B, Schwarzkopf P, Petroff D, Reske AW. Anterior suprascapular nerve block versus interscalene brachial plexus block for shoulder surgery in the outpatient setting: a randomized controlled patient- and assessor-blinded trial. *Reg Anesth Pain Med*. 2017;42:310–318.
3. Reske AW, Schwarzkopf P, Petroff D, Wiegel M. Reply to Dr Stimpson et al. *Reg Anesth Pain Med*. 2017;42:796–797.

Three-Dimensional Thoracic Epidural Educational Model

Accepted for publication: June 25, 2017.

To the Editor:

We read with interest the article entitled, "Use of 3-Dimensional Printing to Create Patient-Specific Thoracic Spine Models as Task Trainers," by Jeganathan et al,[1] introducing the concept of "homemade, low-cost, patient-specific, and high-fidelity ultrasound guidance simulators for resident training in thoracic epidurals using 3-dimensional printing technology."

The combination of high-resolution computed tomography scan data, computer-aided design, and 3-dimensional (3D) printing have led to a revolution in our ability to imagine and build anatomically based teaching tools. Over the past 2 years, we have also used 3D printing and other additive manufacturing techniques to develop an educational model for thoracic epidural training. We presented our work at the 42nd Annual Regional Anesthesiology and Acute Pain Medicine meeting of the American Society of Regional Anesthesiology and Pain Medicine (April 6–8, 2017, in San Francisco, California).

Using stereolithography files based on computed tomography scan data (available free online at https://grabcad.com/), we 3D printed a human thoracic spine from levels T7 to T11. We then 3D printed a simple mold to cast a simulated ligamentum flavum from silicone rubber (https://www.smooth-on.com/product-line/oomoo/). We secured the ligament in the spinal canal with a bubble tea straw, attached the model

© 2017 American Society of Regional Anesthesia and Pain Medicine

Copyright © 2017 American Society of Regional Anesthesia and Pain Medicine. Unauthorized reproduction of this article is prohibited.

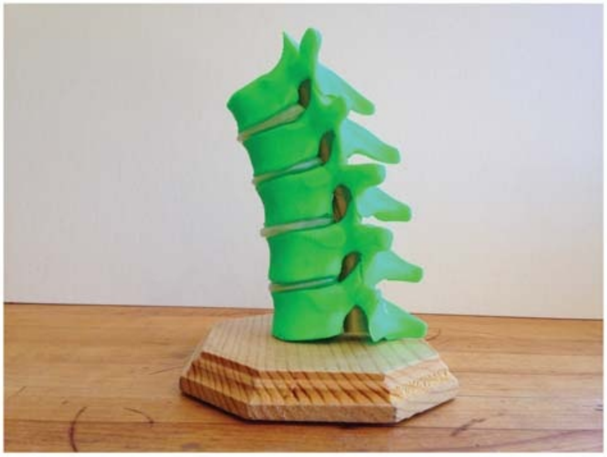

FIGURE 1. Three-dimensionally printed thoracic epidural model.

to a sturdy base, and submerged it in ballistic gel to create the final layer of soft tissue. Ballistic gel is a 12:1 mixture of water and gelatin developed by the military to simulate the density and viscosity of soft tissue (Figs. 1, 2).

Our goal was to make this model low cost, easy to fabricate, and available globally. To that end, we published our design files and an instruction manual on Thingiverse, an open-source community Web site for 3D printing. Our 3D thoracic epidural model can be found at https://www.thingiverse.com/thing:1855444. Cost of materials is approximately $40, and approximate building time is less than 2 days. By comparison, Jeganathan et al describe cost of materials as approximately $400 and time to develop of “less than 4 days.”

This project was completed using a 3D printer and other resources at a university Makerspace. For those without access to a 3D printer, design files can be uploaded to one of several printing hubs and ordered online (https://www.3dhubs.com/).

Our regional anesthesia team places approximately 15 to 20 thoracic epidural catheters per week. We use the model to introduce residents to epidural technique at the beginning of their regional anesthesia rotation. We are currently conducting a survey of resident satisfaction with visual and tactile fidelity of the model and its overall usefulness as a teaching tool. Ultrasound fidelity is excellent.

We commend Jeganathan et al for their excellent work. Their article describes one of the first projects in what promises to be an exciting wave of educational tools based on 3D printing and computer-aided design. We invite others interested in 3D printing to visit our link, build a model for themselves, and modify the design to fit their practice. We are excited about the open-source nature of this project and the possibility of global collaboration to continually improve it.

Martha Claire Johnson, MD
Department of Anesthesiology
and Pain Medicine
University of Washington
Seattle, WA

Sasha Portnova, BS
Matthew Lester, BS
Department of Mechanical Engineering
University of Washington
Seattle, WA

The authors declare no conflict of interest.

REFERENCE

1. Jeganathan J, Baribeau Y, Bortman J, et al. Use of 3-dimensional printing to create patient-specific thoracic spine models as task trainers. *Reg Anesth Pain Med*. 2017;42:469–474.

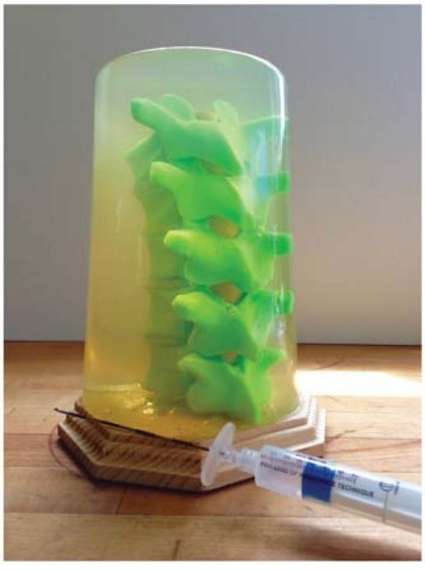

FIGURE 2. Thoracic epidural model with ballistic gel.

Erector Spinae Plane Blocks Provide Analgesia for Breast and Axillary Surgery
A Series of 3 Cases

Accepted for publication: July 23, 2017.

To the Editor:

Excellent acute pain management after breast surgery is critical for patient satisfaction and timely discharge from the recovery unit. Patients undergoing breast surgery have multiple risk factors for postoperative nausea and vomiting (PONV). Furthermore, inadequately treated acute postoperative pain following breast surgery is associated with development of chronic pain.[1] Paravertebral blocks (PVBs) have been demonstrated to significantly improve postoperative analgesia and decrease PONV.[2] However, they are associated with complications including hypotension, neuraxial/intravascular injection, neuraxial hematoma, and pleural puncture.[3] Despite the relatively low complication rates for ultrasound-guided PVB,[4] the risks may outweigh the benefits in patients who would poorly tolerate these complications.

The recently described ultrasound-guided erector spinae plane block (ESPB) has been described for treating chronic thoracic pain and acute postthoracotomy pain, as well as providing abdominal analgesia after bariatric surgery.[5,6] There have been no reports of ESPB for axillary analgesia. We

© 2017 American Society of Regional Anesthesia and Pain Medicine

Copyright © 2017 American Society of Regional Anesthesia and Pain Medicine. Unauthorized reproduction of this article is prohibited.
