# Supplementary material for: A do-it-yourself 3D-printed thoracic spine model for anesthesia resident simulation
Source: PLoS One. 2020 Mar 11;15(3):e0228665. doi: 10.1371/journal.pone.0228665 (PMC7065759; doi:10.1371/journal.pone.0228665)
Supplement: Attachment — Submitted filename: Rebuttal letter.docx [file pone.0228665.s003.docx]

Dear Dr. Meyer, Dr. Mashari, and Reviewer #2,

Thank you for the time you invested in reviewing our manuscript. We appreciate your helpful and insightful comments. We have learned a lot in the process of these revisions, and are even more excited about the future of 3D-printed models in anesthesia education.

We will go through the comments and recommendations point by point.

We are uploading our data to PlosOne as supporting files.

Additional Editor Comments

1. We have reviewed the models described by Jeganathan and Mashari. We have discussed the similarities and unique properties of our model.

2. We have made several models and they are currently in use in the University of Washington Department of Anesthesiology and have also been used for projects in the Department of Mechanical Engineering. To date, there have been 1550 downloads of our design files and manual from Thingiverse, but we have no data on how challenging this would be for “true rookie.” Based on our personal experience (2 of the authors were rookies at the start of the project), we believe that anyone with interest and familiarity with 3D printing can make the model. We have addressed this further in the paper.

3. We added titles to Figures 3-5.

4. We did not include supraspinous or interspinous ligaments in the model because they are not encountered in the standard thoracic epidural approach, which is paramedian.

5. It may be possible to make the entire model of compostable materials, but this would take some thought and design exploration. The current spine, made of PLA, could be recycled or composted in an industrial composting facility, but this is not commonly done.

Reviewer #1 general comments:

Thank you for your comments.

We have posted our files to github.

Reviewer #2 general comments:

Thank you for your comments.

Thank you for pointing out the biggest limitation of our study: lack of a comparator.

We have aimed to address this throughout the paper.

Reviewer #2 specific comments:

Pg 2 ln 43 – We removed the term “greengrocer” from the manuscript.

Pg 3 ln 58 – We eliminated some of the information on history and added information about the advantages of 3D printing in medical education. We address this further in the Discussion.

Pg 3 ln 68 – We simplified this paragraph and put most of this information in the Methods.

Pg 4 ln 75 – We posted our files to github in addition to Thingiverse.

Pg 4 ln 81 – We used an open-source model of the thoracic spine found on GrabCad – we then modified it to print only the 5 thoracic vertebrae and corresponding discs necessary for our model.

Pg 5 ln 115 – We changed the wording here and throughout the paper to clearly state that we assessed the attitudes of anesthesia attendings and residents toward the model.

Pg 5 ln 125 – Same as above.

Pg 5 ln 127 – The actual Likert scale answer choices are found on the y axis of figures 3-5. There are 3 sets of survey questions. The exact wording of the questions for the 1^st^ and 3^rd^ set can be found in Table 1. We summarized the second set of questions as : “Attendings’ perspective on the difficulty of various steps for residents learning thoracic epidural placement for the first time.” The exact wording for these questions is: “In your experience teaching thoracic epidural placement to residents, how difficult is it for the resident to learn: 1. Identifying landmarks..., 2. Determining where…, 3. “How to feel…etc.” We kept this format the same in the revision. We consider it to be accurate and less “busy” for the reader. Current question headings on the left side of Table 1 match the headings on Figs 3-5. Exact wording of questions can also be found in our data files which will be open access.

Pg 6 ln 128 – The exact wording is as follows. We asked the residents: If you had access to a 3D printer would you consider making this model for yourself? We asked the attendings: If you had access to a 3D printer would you consider making this model for resident education? Possible answers: 1 – strongly disagree, 2 – disagree, 3 – neutral, 4 – agree, 5 – strongly agree. Exact wording can be found in our data files which will be open access.

General Methods Questions:

Was the experience with the model standardized? Ie were specific instructions given? Were the proctors present during usage, were they the same proctors, etcs? How much time was spent with model?

We rewrote the Methods section to more clearly state the details of the protocol for practice with the model.

- There is no comparator group or control group. There is no anchoring refence for the survey questions, ie they felt positively about it, but more so than watching a lecture or another traditional method?. How would the model compare to a simple 1 hour didactic or small group PBLD on epidurals or simple scanning on a live model... etc? Generally, any time you give something to people in additional educational time, people feel positively. However, the questions remains, is this Better than existing methods of teaching. This study does not answer that question I think.

We addressed this limitation in the Discussion.

Pg 8 ln 160 – We corrected these typos.

Pg 9 ln 181 –Ultrasound of the model compares favorably to that of an actual person. See images from NYSORA website below:

Transverse View

Paramedian View

<https://www.nysora.com/techniques/neuraxial-and-perineuraxial-techniques/spinal-sonography-and-applications-of-ultrasound-for-central-neuraxial-blocks/>

We would be happy to contact NYSORA to obtain rights to publish these images if necessary.

Pg 10 ln 212 - Correct. This paper does not address whether or not practice with the model improves performance. This could be a done in a future study. However, it would be difficult to do. How do you measure performance? Say you use “time to successful completion of thoracic epidural procedure.” There would be significant variability in the results based on resident factors (novice residents arrive with varied levels of manual skill) and patient factors such as body habitus, normal variation in spine anatomy, and patient temperament. All of these would need to be controlled for. But it could be done.

Pg 10 ln 220 – We address this issue in the Discussion.

Overall discussion comments:

We rewrote the manuscript to better address the limitations of the study.

We also discussed the unique properties of 3D printing that make it ideal for fabrication of medical education models.

Figure 3,4,5 – The exact question wording is in the legend. It would be too busy to place on the x axis.

We do not feel strongly that a comparison ultrasound of a human is necessary for publication.

Our goal with the US image is to show that you can clearly see the ligamentum flavum and boney landmarks. However, if PlosOne editors and reviewers would like us to add comparison images we would be happy to do this.

Best Regards,

Martha C. Johnson MD

Clinical Associate Professor

Department of Anesthesiology and Pain Medicine

University of Washington

Seattle, WA 98195

Tel 206 931 9885
